# 

**Published:** 1876-10

**Authors:** 


ESTABLISHED IN 1855.

Volume XIV | OCTOBER—1876 | Number 7.

S.G.O. INDEXED

### ATLANTA

### Medical and Surgical Journal.

MONTHLY: THREE DOLLARS PER ANNUM, IN ADVANCE.

EDITOR:

J. G. WESTMORELAND, M.D.

*Professor Materia Medica in Atlanta Medical College.*

ASSOCIATE EDITOR:

R. W. WESTMORELAND, M.D.

*Assistant Demonstrator and Prosector of Anatomy in Atlanta Medical College.*

DUNLOP & DICKSON, PUBLISHERS.

*PAX ET SCIENTIA, SED VERITAS SINE TIMORE.*

ATLANTA, GEORGIA:

*DUNLOP & DICKSON, BOOK AND JOB PRINTERS.*

1876.

